# Testosterone therapy increases the anticoagulant potential in men with opioid-induced hypogonadism: a randomized, placebo-controlled study

**DOI:** 10.1530/EC-22-0455

**Published:** 2023-03-10

**Authors:** Mette Bøgehave, Dorte Glintborg, Jørgen Brodersen Gram, Else-Marie Bladbjerg, Marianne Skovsager Andersen, Johannes Jakobsen Sidelmann

**Affiliations:** 1 Department of Clinical Biochemistry, Hospital South West Jutland, University Hospital of Southern Denmark, Esbjerg, Denmark; 2 Unit for Thrombosis Research, Department of Regional Health Research, University of Southern Denmark, Denmark; 3 Department of Endocrinology, Odense University Hospital, Odense, Denmark; 4 Department of Clinical Research, University of Southern Denmark, Odense, Denmark; 5 OPEN, Open Patient data Explorative Network, Odense University Hospital, Region of Southern Denmark, Odense, Denmark

**Keywords:** testosterone, thrombin generation, blood coagulation, hormone therapy, hypogonadism

## Abstract

**Introduction:**

Hypogonadism is prevalent during opioid treatment, and low testosterone concentrations are associated with cardiovascular disease. The effect of testosterone replacement therapy (TRT) on the coagulation system in men with hypogonadism is not clarified. We investigate the effects of TRT on the tissue factor (TF) and contact activation pathways of coagulation in opioid-treated men.

**Materials and methods:**

This was a double-blinded, placebo-controlled study in 37 men with total testosterone < 12 nmol/L randomized to 24 weeks of testosterone injections (*n* = 17) or placebo (*n* = 20). Variables of the coagulation system were analysed at baseline and after 24 weeks. Measurements included the TF pathway (endogenous thrombin potential (ETP) and peak thrombin), the contact activation pathway (endogenous kallikrein potential (EKP) and peak kallikrein), coagulation factors (FVII, FX, prothrombin, and FXII), and inhibitors (tissue factor pathway inhibitor (TFPI), protein C, protein S, antithrombin, and C1 esterase inhibitor (C1inh)). Between-group differences at 24 weeks were determined with analysis of covariance. Within-group changes in TRT and placebo were analysed with paired *t*-test.

**Results:**

Between-group differences at 24 weeks were observed for ETP (*P* = 0.036), FVII (*P* = 0.044), FX (*P* = 0.015), prothrombin (*P* = 0.003), protein C (*P* = 0.004), and protein S (*P* = 0.038). Within the TRT group, ETP, peak thrombin, FVII, FX, prothrombin, TFPI, protein C, FXII, and C1inh decreased and protein S increased (all *P* < 0.05). Within the placebo group, coagulation outcomes were unchanged.

**Conclusion:**

TRT affects the coagulation system in an anticoagulant direction through suppressed TF pathway in men with opioid-induced hypogonadism.

## Introduction

Opioid medications are widely used to treat chronic non-cancer pain (1). Male hypogonadism, characterized by low concentrations of testosterone and luteinizing hormone (LH), is one of the most well-described hormonal adverse effects of opioid treatment (2). The risk of hypogonadism ranges from 19 to 86% in men treated with opioids, depending on the treatment duration, the dosage of opioids, and the applied threshold concentration for total testosterone used to define hypogonadism (3). Low testosterone is associated with a non-beneficial cardiovascular risk profile and increased mortality (4, 5). The cardiovascular safety of testosterone replacement therapy (TRT) in men with reduced testosterone concentrations is, however, controversial (6). TRT improves body composition by increasing the lean body mass and decreasing the total fat mass, resulting in a potential beneficial cardiovascular profile (7, 8, 9). TRT, however, may increase cardiovascular risk by lowering subcutaneous fat, concentrations of adiponectin, and HDL cholesterol (10, 11), and studies have demonstrated increased risk of cardiovascular disease (CVD) in men receiving TRT (12, 13, 14, 15). Other studies, however, reported neutral or beneficial effects of TRT on CVD risk (16, 17, 18), but most studies were small and inconclusive. Randomized controlled trials and long-term data on TRT and cardiovascular safety are requested, as recently concluded in a meta-analysis (19).

**Figure 1:**
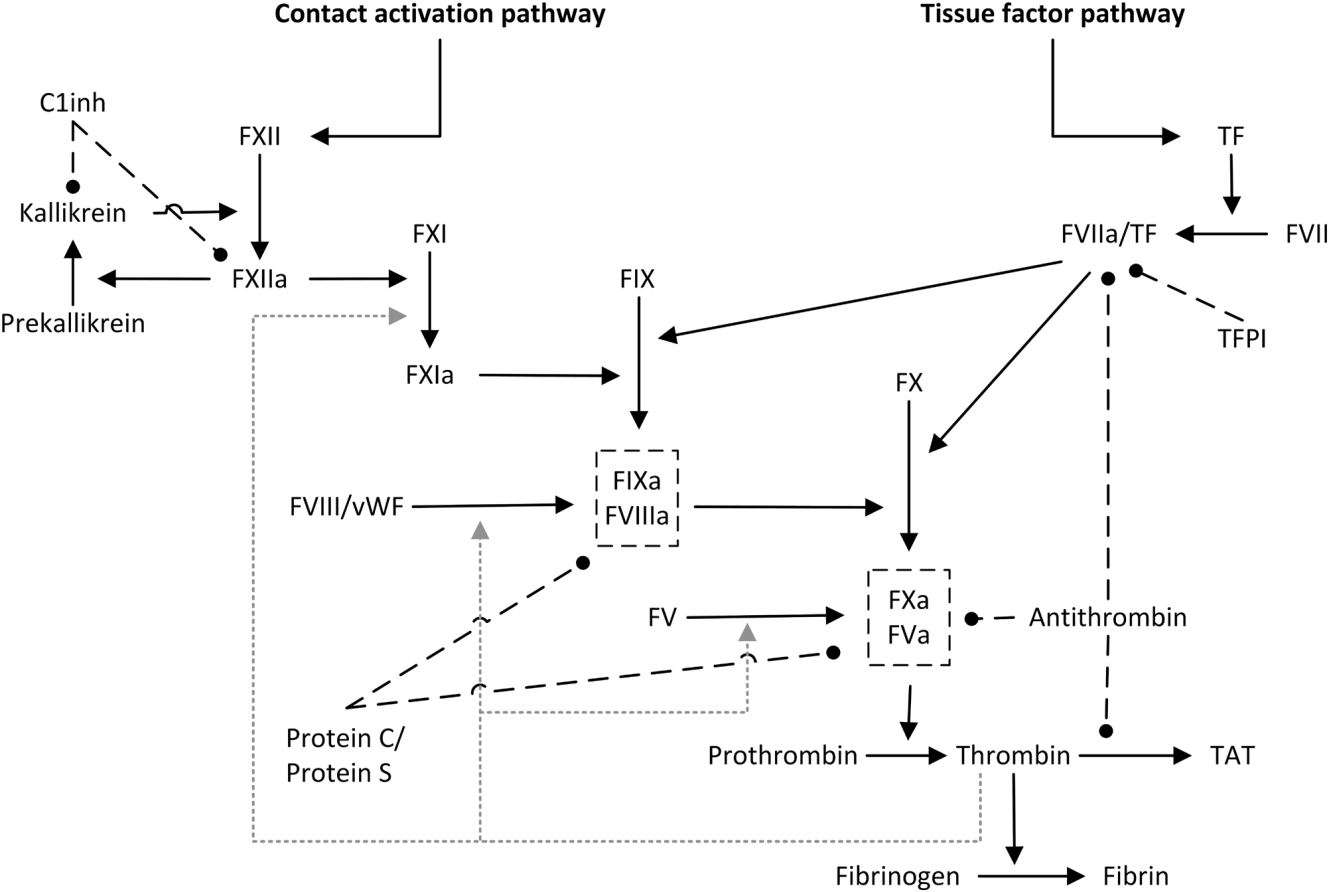
Coagulation system. TF, tissue factor; F, coagulation factor (e.g. FVII); TFPI, tissue factor pathway inhibitor; C1inh, C1 esterase inhibitor; TAT, thrombin–antithrombin complexes; vWF, von Willebrand factor. The letter ‘a’ denotes activated enzymes. Solid lines indicate activation, dashed lines inhibition, and dashed grey lines indicate thrombin-induced feedback activation.

CVD can be caused by atherosclerosis and thrombosis, and alterations in the coagulation system (Fig. 1) contribute significantly to the pathogenesis of thrombosis. It remains unclear whether TRT induces a pro- or anticoagulant state. Reportedly, physiological doses of testosterone to elderly men with hypogonadism induce no significant change in the coagulation system in terms of tissue factor (TF)-induced thrombin generation (20), whereas another study in men without hypogonadism points towards a TRT-induced suppression of coagulation (21). In line with this, reduced thrombin generation, and thus a potential anticoagulant effect of TRT, is reported in men with hypogonadism due to Klinefelter syndrome (22). High doses of testosterone administrated to men without hypogonadism augment the activation of the coagulation system (23). More extreme doses of androgens, as can be seen during anabolic androgenic steroid (AAS) abuse, induce a procoagulant effect related to TF-induced thrombin generation (24). Another study on AAS abuse demonstrated reduced contact-induced kallikrein generation in terms of peak kallikrein concentration and endogenous kallikrein potential (EKP) in current abusers compared with former abusers and controls (25). Conversely, TRT of transgender men (born women) is not associated with a procoagulant state (26) and TRT given to castrated male rats normalizes the castration-induced increase in prothrombin and coagulation factor VII (FVII) concentrations (27). Earlier studies report reduced concentrations of prothrombin in TRT-treated rats (28, 29).

In the present randomized, double-blinded, placebo-controlled intervention study, we investigate in detail the effect of physiological doses of testosterone on the capacity of the TF and contact activation pathways of coagulation and related coagulation factors and inhibitors in opioid-treated men.

## Materials and methods

### Participants and study design

This study is a continuation of a previous study described elsewhere (7). In short, the study is a single-centre, randomized, double-blinded, placebo-controlled study conducted from August 2016 to August 2019 at Odense University Hospital, Denmark. Forty-one men aged >18 years, treated with opioids for non-malignant pain disease, were randomized to receive TRT (testosterone undecanoate (Nebido), 1000 mg i.m.) or identically looking placebo injections. The participants received injections at time of randomization and at 6 and 18 weeks. Inclusion criteria were opioid treatment for at least 3 months at a dosage corresponding to at least 50 mg morphine/day, at least two measures of total testosterone < 12 nmol/L measured in the fasting state between 08:00 and 10:00 in the morning, and LH and prolactin levels within the reference interval. We excluded men wishing to conceive during the trial period, subjected to previous or current testosterone treatment, treated with 5α-reductase inhibitors, oral glucocorticoid steroids, or anticoagulants. We also excluded men with haematocrit > 53%, previous or ongoing malignant disease, prostate-specific antigen > 3 ng/dL, abnormal routine blood samples (thyroid-stimulating hormone, ionized calcium, haemoglobin, liver, and kidney function), untreated ischemic heart or respiratory disease, and alcohol or drug abuse.

For various reasons, three participants dropped out during the intervention period (7), and for one participant the blood sampling was incomplete, leaving 17 and 20 participants in the TRT and placebo groups, respectively. The subjects’ characteristics before and 6 months after TRT or placebo are presented in Table 1, showing that body weight, lean body mass, and concentrations of testosterone increase after TRT, whereas the total fat mass is reduced, as previously reported (7).

**Table 1:** Clinical characteristics at baseline and after 24 weeks in men treated with placebo and testosterone replacement therapy.

	Placebo			Testosterone replacement therapy		
Variable	Baseline	24 weeks	*P*	Baseline	24 weeks	*P*
*N*	20	20		17	17	
Age (years)	53 ± 11			54 ± 7		
Weight (kg)	98.7 ± 15.5	99.7 ± 15.5	0.37	100.9 ± 13.2	104.4 ± 14.1	**<0.01**
BMI (kg/m^2^)	32.0 ± 4.1	32.3 ± 4.0	0.56	31.3 ± 3.8	32.5 ± 4.3	**<0.01**
Total fat mass (kg)	33.2 ± 6.1	33.8 ± 6.5	0.26	33.0 ± 7.5	31.6 ± 7.8	**<0.01**
Fat percentage (%)	34.9 ± 2.8	35.5 ± 3.4	0.24	33.8 ± 5.1	31.6 ± 4.4	**<0.01**
Lean body mass (kg)	59.7 ± 9.3	59.3 ± 9.8	0.57	61.5 ± 7.7	65.2 ± 7.3	**<0.01**
Total testosterone (nmol/L)^a^	7.4 (5.1; 9.6)	7.7 (5.2; 8.5)	0.46	6.8 (5.2; 9.5)	19.4 (14.3; 26.8)	**<0.01**
Sex hormone-binding globulin (nmol/L)^a^	32.2 (24.5; 44.5)	31.5 (20.7; 51.2)	0.48	41.9 (24.5; 51.7)	36.9 (24.6; 45.9)	**<0.01**
Bioavailable testosterone (nmol/L)^a^	3.5 (2.1; 4.4)	3.0 (1.9; 4.1)	0.40	2.7 (2.0; 3.8)	8.6 (8.1; 12.4)	**<0.01**
Total cholesterol (mmol/L)	4.8 ± 1.0	4.8 ± 1.2	0.71	4.5 ± 0.9	4.3 ± 0.9	0.30
LDL (mmol/L)	2.8 ± 1.0	2.7 ± 1.0	0.38	2.6 ± 0.8	2.5 ± 0.7	0.41
Triglycerides (mmol/L)^a^	1.6 (1.1; 2.9)	1.8 (1.2; 3.1)	0.53	1.5 (1.0; 2.2)	1.8 (1.0; 2.3)	0.60

Data presented as mean ± s.d. were compared at baseline and 24 weeks with a paired *t*-test. Subjects’ characteristics were previously reported in Glintborg *et al* (7). ^a^Data presented as median (25 and 75 percentiles) were compared with a Wilcoxon test. Significant differences are highlighted in bold. BMI, body mass index; LDL, low-density lipoprotein cholesterol.

### Ethics

The study was planned in May 2015 and approved by The Regional Scientific Ethical Committee of Southern Denmark (S-20150004) and the Danish Health and Medicines Agency. Written informed consent was obtained from all participants. The trial was declared in www.clinicaltrials.gov (NCT02433730).

### Blood sampling

The collection and handling of blood specimens followed the G41 guideline from Clinical and Laboratory Standards Institute (CLSI) (30) with specific focus on the recommendations for collection, transport, and processing of blood specimens for testing plasma-based coagulation assays, as detailed in the H21-A5 guideline from CLSI (31). In brief, fasting blood was collected from an antecubital vein in evacuated 2.7 mL tubes containing 0.109 mol/L sodium citrate (Vacutainer 9NC, Becton Dickinson, Plymouth, UK). Samples were obtained at baseline and after 6 month (24 week) intervention. Platelet-poor plasma was collected after centrifugation for 20 min at 2000 × ***g***. The citrate-stabilized plasma specimens were stored at −80°C in tightly capped cryotubes (Sarstedt, VWR-Bie & Berntsen, Søborg, Denmark). Before analysis, the samples were thawed for 5 min at 37°C, kept at room temperature, and analysed within 1 h.

### Biochemical analyses

Coagulation assays were performed at the Unit for Thrombosis Research, Hospital of South West Jutland, Esbjerg, Denmark.

### Thrombin and kallikrein generation

TF-induced thrombin generation was analysed by the calibrated automated thrombin generation assay (Thrombinoscope BV, Maastrict, the Netherlands) using 5 pM TF and the Fluoroskan Ascent microplate fluorometer (Thermo Fisher Scientific) (32). In brief, thrombin was generated by mixing 80 µL plasma with 20 µL fluorogenic substrate-calcium chloride and 20 µL activating reagent with a final concentration of 5 pM TF and 4 µM phospholipids. The Thrombinoscope BV software was used for the calculation of various measures of thrombin generation: the lag time of the thrombin formation process (lag time, min), the time until the peak thrombin concentration was reached (time to peak, min), the peak thrombin concentration (peak, nmol/L), and the endogenous thrombin potential (ETP, nmol/L × min), representing the total amount of thrombin formed.

Contact-induced kallikrein generation was determined as described previously (33). In brief, undiluted citrate plasma was activated with silica (Sigma) diluted in activated partial thromboplastin time reagents (Triolab, Brøndby, Denmark), and FXII-dependent kallikrein generation was measured with the fluorogenic substrate Boc-Leu-Lys-Arg-AMC (Bachem, Bubendorf, Switzerland). Affinity-purified human kallikrein (Enzyme Research Laboratories, Swansea, UK) was used as a calibrator. Fluorescence was read using the Fluoroskan Ascent microplate fluorometer. The Thrombinoscope software was used to generate kallikrein formation curves. The curves display kallikrein generation lag time (lag time, min), peak kallikrein generation (peak, nmol/L), and area under the curve (EKP, nmol/L × min), representing the total amount of kallikrein formed.

### Coagulation factors and inhibitors

The plasma protein concentration of coagulation factor XII (FXII) was determined with enzyme-linked immunosorbent assay (ELISA), as described previously (34). Plasma coagulation factor X (FX), FVII, and prothrombin were determined by clotting assays employing the ACLTOP350 Coagulation Analyzer, the HemosIL RecombiPlasTin 2G, and FX-, FVII-, and prothrombin-deficient plasmas. Analyzer, calibrators, and factor diluents were all from Instrumentation Laboratory, ILS Denmark, Lillerød, Denmark. Plasma antithrombin was determined by an automated chromogenic assay (STA-Stachrom AT III, Stago, Asnieres-sur-Seine, Paris, France). Plasma protein C activity was determined by an automated chromogenic assay (Protein C, HemosIL, Instrumentation Laboratory). Plasma-free protein S antigen was determined using an immune-turbidimetric assay kit (Free Protein S, HemosIL, Instrumentation Laboratory). The assays were performed on the ACLTOP350 Coagulation Analyzer. Plasma tissue factor pathway inhibitor (TFPI) antigen was determined using an ELISA (Human TFPI, Quantikine ELISA, R&D Systems Inc) using a microplate reader (Sunrise, Tecan Trading AG, Basle, Switzerland). The protein concentration of C1 esterase inhibitor (C1inh) was determined using N antiserum against human C1inh, buffers, and reagents, employing the BN II analyzer (all from Siemens Healthcare Diagnostics).

### Statistical analysis

Variables of the haemostatic system as outcome measures were not defined in the original study (7), which was planned in 2015, and the power of the study was determined by the effect of TRT on lean body mass. Likewise, no studies examined the effect of TRT on variables of the haemostatic system in opioid-induced hypogonadism, and a power calculation was not performed. Thus, the present study should be regarded as a pilot study. However, in a *post hoc* power calculation, we selected ETP as the main outcome variable. The number of patients included in the study was sufficient to detect a 200 nmol/L × min difference in ETP between TRT and placebo groups, at a significance level of 5%, a power of 80%, and a standard deviation (s.d.) of 225 nmol/L × min. This difference in ETP is comparable to the difference observed in a previous study of TRT-treated men with Klinefelter syndrome (22). The present analysis is an efficacy analysis with the aim to determine biological effects of TRT, and data were analysed according to a per-protocol analysis on completers only. We did not include an intention-to-treat (ITT) analysis, because the coagulation variables were only measured in study completers.

Baseline values were compared between the TRT and placebo groups using an unpaired *t*-test. Between-group differences (TRT vs placebo) at 24 weeks were determined with an analysis of covariance, adjusted for baseline values, due to the randomized study design where the focus was on the size of treatment effects. Within-group changes (in TRT and placebo groups, respectively) were analysed with a paired *t*-test. TFPI was logarithmically transformed to meet the normal distribution assumption. Results are presented as mean (or geometric mean for TFPI) values and s.d.. A significance level of 0.05 was used throughout the study. The Statistical Package for Social Sciences (SPSS) program version 28 (IBM) was used for all statistical analyses.

## Results

**Figure 2:**
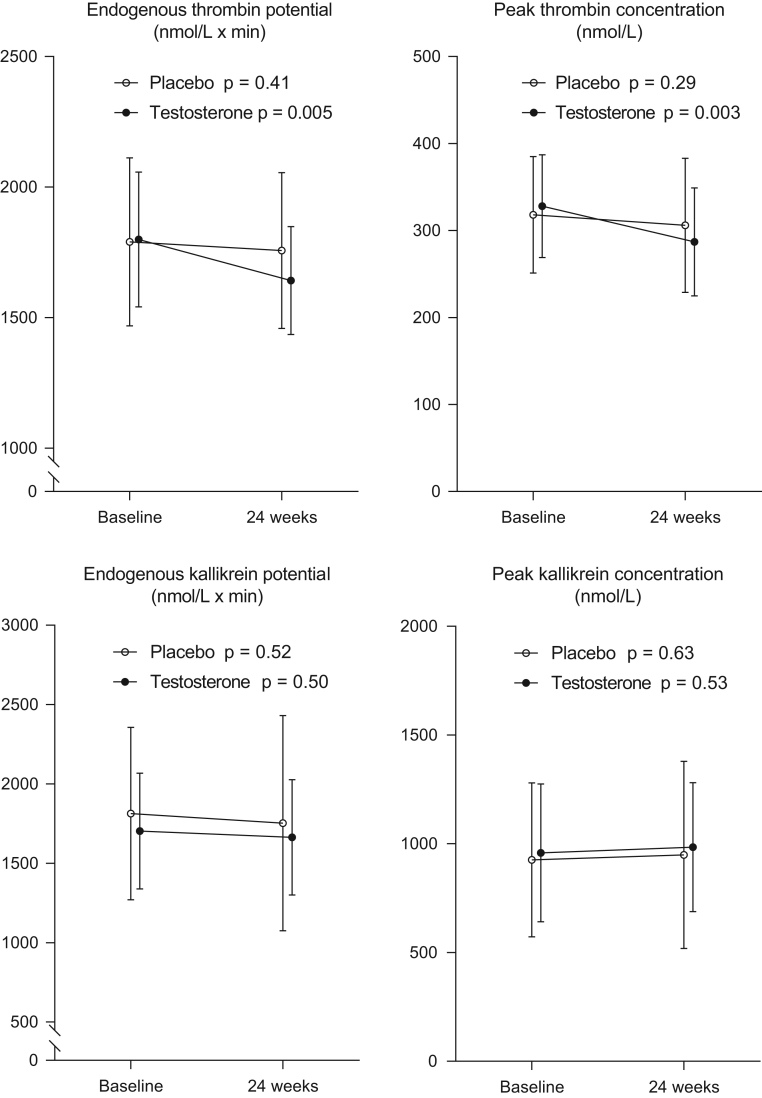
Measures of generation of thrombin and kallikrein generation. Endogenous thrombin potential, peak thrombin concentration, endogenous kallikrein potential, and peak kallikrein concentration at baseline and after 24 weeks of testosterone replacement therapy or placebo. Results are presented as mean ± s.d.

Measures of thrombin generation, kallikrein generation, coagulation factors, and inhibitors at baseline and after 24 weeks of placebo and TRT are shown in Table 2 and Fig. 2. No significant between-group differences were detected in baseline values.

**Table 2:** Coagulation factors and inhibitors at baseline and after 24 weeks in men treated with placebo and testosterone replacement therapy.

	Placebo			Testosterone replacement therapy		
Variable	Baseline	24 weeks	*P*	Baseline	24 weeks	*P*
*N*	20	20		17	17	
Coagulation factor VII (%)	126 ± 23	120 ± 28	0.13	118 ± 21	101 ± 21	**<0.001**
Coagulation factor X (%)	119 ± 20	118 ± 19	0.87	119 ± 19	110 ± 13	**<0.01**
Prothrombin (%)	111 ± 14	109 ± 12	0.22	106 ± 12	97 ± 9	**<0.01**
Protein C (%)	128 ± 29	127 ± 31	0.70	120 ± 26	107 ± 20	**<0.01**
Free protein S (%)	111 ± 14	110 ± 16	0.53	107 ± 19	112 ± 16	**<0.01**
Tissue factor pathway inhibitor (ng/mL)^a^	31.1 ± 1.25	30.1 ± 1.27	0.33	30.1 ± 1.25	28.3 ± 1.26	**<0.05**
Antithrombin (%)	100 ± 10	100 ± 8	0.97	94 ± 12	91 ± 13	0.08
Coagulation factor XII (%)	134 ± 29	131 ± 29	0.47	136 ± 28	123 ± 27	**<0.01**
C1 esterase inhibitor (%)	88 ± 20	83 ± 21	0.10	91 ± 26	84 ± 25	**<0.05**

Data presented as mean ± s.d. were compared at baseline and 24 weeks with a paired *t*-test. Significant differences are highlighted in bold.^a^Tissue factor pathway inhibitors were logarithmically transformed before analysis (geometric mean ± s.d.).

In the TF pathway of coagulation, we observed between-group differences at 24 weeks for ETP (*P* = 0.036), FVII (*P* = 0.044), FX (*P* = 0.015), prothrombin (*P* = 0.003), protein C (*P* = 0.004), and protein S (*P* = 0.038). No between-group differences at 24 weeks were observed for peak thrombin concentration (*P* = 0.10), TFPI (*P* = 0.41), or antithrombin (*P* = 0.053).

Within the TRT group, ETP decreased (1642 ± 207 vs 1799 ± 258 nmol/L × min, *P* = 0.005) and peak thrombin concentration decreased (287 ± 62 vs 328 ± 59 nmol/L, *P* = 0.003) (Fig. 2). Within the placebo group, ETP and peak thrombin concentration were not significantly different (1757 ± 299 vs 1790 ± 322 nmol/L × min, *P* = 0.41 and 306 ± 77 vs 318 ± 67 nmol/L, *P* = 0.29, respectively) (Fig. 2). Furthermore, TRT was associated with significant decreases from baseline to 24 weeks in plasma levels of FVII, FX, and prothrombin and in the coagulation inhibitors TFPI and protein C. Free protein S increased significantly (Table 2).

The variables of the contact activation pathway (EKP (*P* = 0.92), peak kallikrein concentration (*P* = 0.96), FXII (*P* = 0.051), and C1inh (*P* = 0.64)) were comparable between TRT and placebo at 24 weeks. Within the TRT group, EKP and peak kallikrein concentration were not significantly different (1664 ± 364 vs 1703 ± 365 nmol/L × min, *P* = 0.50 and 984 ± 297 nmol/L vs 958 ± 317 nmol/L, *P* = 0.53, respectively). In the placebo group, no significant changes were found after 24 weeks in EKP (1753 ± 678 vs 1813 ± 543 nmol/L × min, *P* = 0.52) or in peak kallikrein concentration (949 ± 430 vs 926 ± 354 nmol/L, *P* = 0.63) (Fig. 2). We found significantly reduced concentrations of FXII and C1inh from baseline to 24 weeks in the TRT group but not in the placebo group (Table 2).

## Discussion

This study represents the largest randomized, placebo-controlled study addressing the effect of physiological doses of testosterone on the coagulation system in men with hypogonadism. We present a potential anticoagulant effect of TRT by demonstrating decreased TF-induced thrombin generation, decreased activity of the vitamin K-dependent coagulation factors, and protein C and increased protein concentration of free protein S. TRT compared to placebo did not affect the contact system proteins FXII and C1inh and did not translate into changes in the kallikrein generation capacity of the contact system. Thus, our findings support that TRT reduces the coagulation potential of the TF pathway of coagulation.

The influence of TRT on measures of thrombin generation was addressed in a previous controlled study, where no changes in peak thrombin concentration or ETP were found after 52 weeks of testosterone undecanoate injections in 13 elderly men with low baseline testosterone concentrations (20). The modest study population, a mean age of 69 years, and the age-related baseline low testosterone levels may explain these discrepancies. The 37 men included in the present study had a mean age of 53 years and medication-induced decreased testosterone concentrations. Furthermore, the duration between the latest injection of testosterone and blood sampling was 12 weeks in the former study (20) and 6 weeks in the present study, indicating that the effect of TRT could decline in 12 weeks. This could point towards an acute and not persistent effect of testosterone on thrombin generation.

The thrombin generation method applied in the present study involves the TF pathway of coagulation encompassing FVII, FX, and prothrombin, and FX and prothrombin are here the major determinants of thrombin generation (35). Accordingly, we demonstrate that TRT is associated with a significant decrease in clotting activities of FX and prothrombin, resulting in a decrease in ETP. A study of transgender men receiving TRT demonstrated decreased prothrombin concentrations in line with our findings (26). The decrease in FVII and prothrombin induced by TRT is in accordance with previous findings in animal studies (27, 28, 29, 36, 37, 38). The influence of TRT on FX has not been addressed in other studies.

Studying the effect of TRT on the inhibitory components of the TF pathway, we demonstrate that TRT is associated with a decrease in TFPI and protein C and an increase in protein S and has no significant effect on antithrombin. However, only protein C and protein S were significantly different from the placebo group. Corresponding to our findings, previous studies report a reduction in protein C (23), an increase in free protein S (26), and no effect on TFPI and antithrombin (20, 22) after testosterone therapy.

The mechanism behind the decreased thrombin formation through the TF pathway in the testosterone-treated men is not elucidated in the present study. Speculatively, a direct effect of testosterone through the androgen receptor on liver protein synthesis is possible, as we demonstrate decreased levels of liver-derived coagulation factors and inhibitors among testosterone-treated men. In fact, only the functional level of the vitamin K-dependent coagulation factors and inhibitors were reduced by TRT compared with placebo, indicating an effect of testosterone supplementation on vitamin K metabolism. A previous animal study has suggested that androgens may affect the components of the vitamin K cycle, resulting in reduced synthesis of the vitamin K-dependent coagulation factors and inhibitors (29). Regarding free protein S, more than half of plasma protein S circulates in complex with C4b-binding protein, and studies have indicated that TRT reduces the concentration of this plasma protein (23). Thus, a TRT-induced altered balance between free and total protein S may increase concentrations of free protein S.

This is the first study to address the potential effect of TRT on the capacity of the contact activation pathway of coagulation. Although we observed a decrease in FXII, in line with previous findings (21), and in C1inh, the turnover of the contact activation pathway was not affected by TRT as no overall changes in the kallikrein generation capacity was observed, indicating an equivalent testosterone-induced reduction in the plasma concentrations of the two proteins.

The clinical impact of the observed effects on thrombin generation remains to be elucidated. Recently, increased measures of thrombin generation were associated with a procoagulant state and increased risk of deep venous thrombosis, thrombosis recurrence, as well as acute ischemic stroke (39). Hence, our documented reduction in ETP could indicate an antithrombotic effect of testosterone. TRT may affect various organs and processes, and TRT has a positive impact on body composition with reduced fat mass and fat percentage and increased lean body mass (8, 9, 10), as confirmed in this study population (Table 1). Excessive erythrocytosis (40) and elevated hematocrit values, however, occur in up to 40% of patients receiving androgen treatment (41), leading to increased blood viscosity (42), which potentially augments blood-flow resistance and hence causes a prothrombotic effect. It is tempting to speculate that the presently observed decrease in thrombin generation potential and the beneficial effects on body composition counterbalance the prothrombotic increase in blood viscosity induced by TRT.

The strength of the present study is the randomized, double-blinded, and placebo-controlled design. The study is considered a pilot study as the power calculation of the original study was based on lean body mass; hence, the present study might be underpowered to study other outcomes. The relatively low number of study participants increases the risk of type II errors. Thus, we cannot exclude an effect of TRT on all liver-derived coagulation proteins and not only the vitamin K-dependent proteins. We did not perform an ITT analysis, but only three study participants were lost to follow-up, and therefore we do not expect a significant difference between ITT and per-protocol analyses. The study participants had relative hypogonadism with testosterone concentrations < 12 nmol/L; hence the observed effect on thrombin generation is expected to be greater in men with absolute hypogonadism.

In conclusion, TRT has a suppressive effect on the TF pathway of coagulation as we demonstrate a significant reduction in ETP and in the functional levels of coagulation factors and inhibitors and an increase in free protein S in men with opioid-induced decreased testosterone concentrations.

## Declaration of interest

None declared.

## Funding

The Karola Jørgensens Forskningsfond provided financial grant for analysis of data. Bayer provided Nebido® and placebo and the Novo Nordisk Foundation provided financial support, but they were otherwise not involved in the study planning or interpretation of results. The authors would also like to thank the Institute of Clinical Research – University of Southern Denmark for support.

## Authors contribution statement

The authors’ contributions to the present study were as follows: DG, MSA, JBG, and JJS: conceptualization and methodology; MB, EMB, and JJS: data analysis and data interpretation; MB: drafting of the manuscript. All authors were involved in the design of the study, critical revision of the manuscript, and approval of the final manuscript.
